# 

**Published:** 1877-01

**Authors:** 


### ARTICLE IV.

### *Virginia State Dental Association.*

CHARLOTTESVILLE, NOVEMBER 1, 1876.

DEAR SIR—Your attention is respectfully asked to the following "Circular" from the President of our "State Dental Association," announcing the postponement of the meeting for this year, the list of "Standing Committees," and notice of the time and place of meeting for the year 1877.

Very respectfully, your obedient servant,

JOHN W. SCRIBNER, D. D. S.

*Corresponding Secretary.*

---

### CIRCULAR.

FARMVILLE, VA., NOV. 1st, 1876.

*To the Members of the "State Dental Association of Virginia," and the Profession Generally, Residing and Practicing within the State:*

GENTLEMEN.—The Centennial Celebration at Philadelphia, and the several meetings of dental conventions and associations held in that city during the present year having attracted most, if not all, the members of our State Association, and rendered it improbable, in view of the stringency of the times, that we would have a full or general attendance at our regular annual meeting in December, and for other reasons and considerations, the wisdom and policy of which we recognize and approve, it has been determined by the Executive Committee of the Association to recommend that *no meeting* be held during the present year, A. D., 1876.

We therefore announce to the profession in the State of Virginia, and to the members of our Association, that the annual December meeting for 1876 will not be held ; that the officers, both elected and appointed, will hold over to the next annual meeting, which will be held in the City of Richmond, the second Monday in December, 1877, at which a full and general attendance is desired.

The profession in the State, not now members of our organization, and our professional friends abroad, are earnestly and cordially invited to meet with us on that occasion.

The following are the regular Standing Committees, established by the constitution adopted at the last annual meeting, and as the interest and value of our transactions is so largely dependent upon the reports of these several committees, we cannot too earnestly urge the faithful performance of the duties assigned the gentleman composing them. In this connection we beg to ask the attention of the committees to the following articles of our new and amended Constitution:

ARTICLE 1.—Section 1.—It shall be the duty of each member of a committee to make an individual report, so far as such report can be made, and in case of inability to be present at the meeting at which such report is due, to forward it to the Recording Secretary, from whom it may be obtained by the chairman of each committee respectively at the time of the assembling of the Association.

Section 2d.—Each committee shall report, if practicable, the results of original investigations in their several departments, and also such new matter collected from all sources at their command as may be of interest and profit to the Association.

Section 3d.—The Committee on Operative and Mechanical Dentistry shall thoroughly test and report upon all new models and materials, and upon their physical properties, stating clearly why any particular material or mode of practice should claim attention, and giving tabulated lists of successes and failures, so far as may be attainable."

### REGULAR STANDING COMMITTEES.

*Committee on Publication*—Dr. S. H. Henkel, Staunton, Va.; Dr. L. Sydnor, Salem, Va.; Dr. Frank Harris, Harrisonburg, Va.

*Committee on Operative Dentistry.*—Dr. James F. Thompson, Fredericksburg, Va.; Dr. C. A. Mercer, Richmond, Va.; Dr. G. A. Sprinkel, Culpeper, Va.

*Committee on Mechanical Dentistry.*—Dr. J. Hall Moore, Richmond, Va.; Dr. George Chewning, Fredericksburg, Va.; Dr. E. R. Perron, Lovingston, Va.

*Committee on Dental Education and Literature.*—Dr. James Johnston, Staunton, Va.; Dr. George F. Keesee, Richmond, Va.; Dr. Gutielmus Smith, Louisa C. H., Va.

*Committee on Dental Pathology.*—Dr. George B. Steel, Richmond, Va.; Dr. John W. Scribner, Charlottesville, Va.; Dr. A. C. Smith, Louisa C. H., Va.

*Committee on Physiology, Histology Microscopy and Chemistry.*—Dr. Judd B. Wood, Richmond, Va.: Dr. W. Leigh Burton, Richmond, Va.; Dr. F. A. Jeter, Richmond, Va.

W. W. H. Thackston, *President.*

John W. Scribner, D. D. S., *Cor. Secretary.*

*Alumni Meeting.*—The Annual Meeting of the Alumni Association of the Baltimore College of Dental Surgery, will be held at the College Building, Baltimore, on Friday, March 9th, 1877, commencing at 10 A. M.

*The Thirty-Seventh Annual Commencement* will take place on the evening of the same day, at the Academy of Music. All Graduates of the College, and others interested in Dental Science, are cordially invited to be present.

SAML. J. COCKERILLE, of Class of 1853,
*President.*

WM. B. WISE, of Class of 1874,
*Cor. Secretary.*

### TO READERS AND CORRESPONDENTS.

Communications intended for publication, and exchange journals and papers, should be addressed to the EDITOR OF THE AMERICAN JOURNAL OF DENTAL SCIENCE, 259 N. Eutaw St., Baltimore, Md. All letters relating to the business of the journal, or containing cash remittances, to be directed to Messrs. SNOWDEN & COWMAN. Articles for publication should be received by the editor at least three weeks previous to the first day of the month on which the number in which it is designed for them to appear, is issued.

### MONTHLY SUMMARY.

Cure for Tetanus by Mechanical Measures .......... 426
Is Alcohol a Food? .......... 427
Medical Women .......... 428
Congenital Cleft Palate .......... 429
Chloral Plaster in Neuralgia .......... 429
Irritating Effects of Salicylic Acid .......... 430
On the Local Action of Atropia .......... 431
Deaths from Chloroform .......... 431
Carbolic Acid as a Local Anæsthetic .......... 432
Mastic Chewing .......... 432
A Sensible Precaution .......... 432

☞The American Journal of Dental Science will contain fifty pages of reading matter in each number, making a volume of not less than

*Six Hundred* pages

EXCLUSIVE OF ADVERTISEMENTS

THE

### AMERICAN JOURNAL
OF
### DENTAL SCIENCE.

F. J. S. GORGAS, M. D., D. D. S., Editor.

---

The *Journal* is issued on the first of each month and contains not less than *fifty* pages of reading matter in each number, exclusive of advertisements, making a volume of *six hundred* pages per annum.

In each number will be found Original Communications, Correspondence, Selected Articles, Monthly Summary, Bibliographical Notices and Editorial Matter, and every effort will be exerted to render the *Journal* worthy of the patronage of the Dental profession.

A generous co-operation is respectfully solicited from the members of the profession; and as the *Journal* has now a printing office of its own, which materially lessens the cost of its publication, it will be issued at the reduced subscription price of

**$2.50 Per Annum in Advance.**

Communications are respectfully solicited on all subjects pertaining to the practice of dentistry, as well as reports of cases occurring in dental practice.

---

### RATES OF ADVERTISING.

| | 1 MO. | 2 MO. | 3 MO. | 6 MO. | 12 MO. |
|---|---|---|---|---|---|
| 1 PAGE. | $15 00 | $22 50 | $30 00 | $50 00 | $100 00 |
| ½ " | 10 00 | 15 00 | 20 00 | 30 00 | 50 00 |
| ¼ " | 7 00 | 11 00 | 15 00 | 20 00 | 30 00 |
| ⅛ " | 5 00 | 8 00 | 11 00 | 15 00 | 20 00 |

All original communications designed for publication, works for review, new instruments for notice, exchanges, &c., should be directed to the editor, 259 N Eutaw St., Baltimore, Md.

All advertisements and subscriptions should be addressed to

SNOWDEN & COWMAN,
*Publishers of the Am. Journal of Dental Science.*
86 W. FAYETTE ST. BET. CHARLES & LIBERTY STS.,
BALTIMORE, MD.

